# Monogenic Diabetes Modeling: *In Vitro* Pancreatic Differentiation From Human Pluripotent Stem Cells Gains Momentum

**DOI:** 10.3389/fendo.2021.692596

**Published:** 2021-07-06

**Authors:** Juan Ignacio Burgos, Ludovic Vallier, Santiago A. Rodríguez-Seguí

**Affiliations:** ^1^ Departamento de Fisiología, Biología Molecular y Celular, Facultad de Ciencias Exactas y Naturales, Universidad de Buenos Aires and Instituto de Fisiología, Biología Molecular y Neurociencias (IFIBYNE), CONICET-Universidad de Buenos Aires, Ciudad Universitaria, Buenos Aires, Argentina; ^2^ Wellcome-Medical Research Council Cambridge Stem Cell Institute and Department of Surgery, University of Cambridge, Cambridge, United Kingdom

**Keywords:** pancreas, beta cell, human, pluripotent stem cell, monogenic, modeling, diabetes, *in vitro* differentiation

## Abstract

The occurrence of diabetes mellitus is characterized by pancreatic *β* cell loss and chronic hyperglycemia. While Type 1 and Type 2 diabetes are the most common types, rarer forms involve mutations affecting a single gene. This characteristic has made monogenic diabetes an interesting disease group to model *in vitro* using human pluripotent stem cells (hPSCs). By altering the genotype of the original hPSCs or by deriving human induced pluripotent stem cells (hiPSCs) from patients with monogenic diabetes, changes in the outcome of the *in vitro* differentiation protocol can be analyzed in detail to infer the regulatory mechanisms affected by the disease-associated genes. This approach has been so far applied to a diversity of genes/diseases and uncovered new mechanisms. The focus of the present review is to discuss the latest findings obtained by modeling monogenic diabetes using hPSC-derived pancreatic cells generated *in vitro*. We will specifically focus on the interpretation of these studies, the advantages and limitations of the models used, and the future perspectives for improvement.

## Introduction

Diabetes mellitus (DM) is characterized by pancreatic *β* cell loss and chronic hyperglycemia. Type 1 diabetes (T1D) is caused by the autoimmune reaction against *β* cells (1), and Type 2 diabetes originates from insulin resistance and *β* cell overload (2–4). In addition, rarer monogenic forms of diabetes account for approximately 1–5% of diabetes cases, depending on the population studied (5, 6). Over 30 subtypes of monogenic diabetes have been identified to date, each having a characteristic phenotype and a specific pattern of inheritance (6, 7). The identification of genes implicated in the pathogenesis of monogenic diabetes, including components of the insulin secretory pathway and transcription factors, has provided important insights into human pancreas and *β* cell development and function.

**Figure 1:**
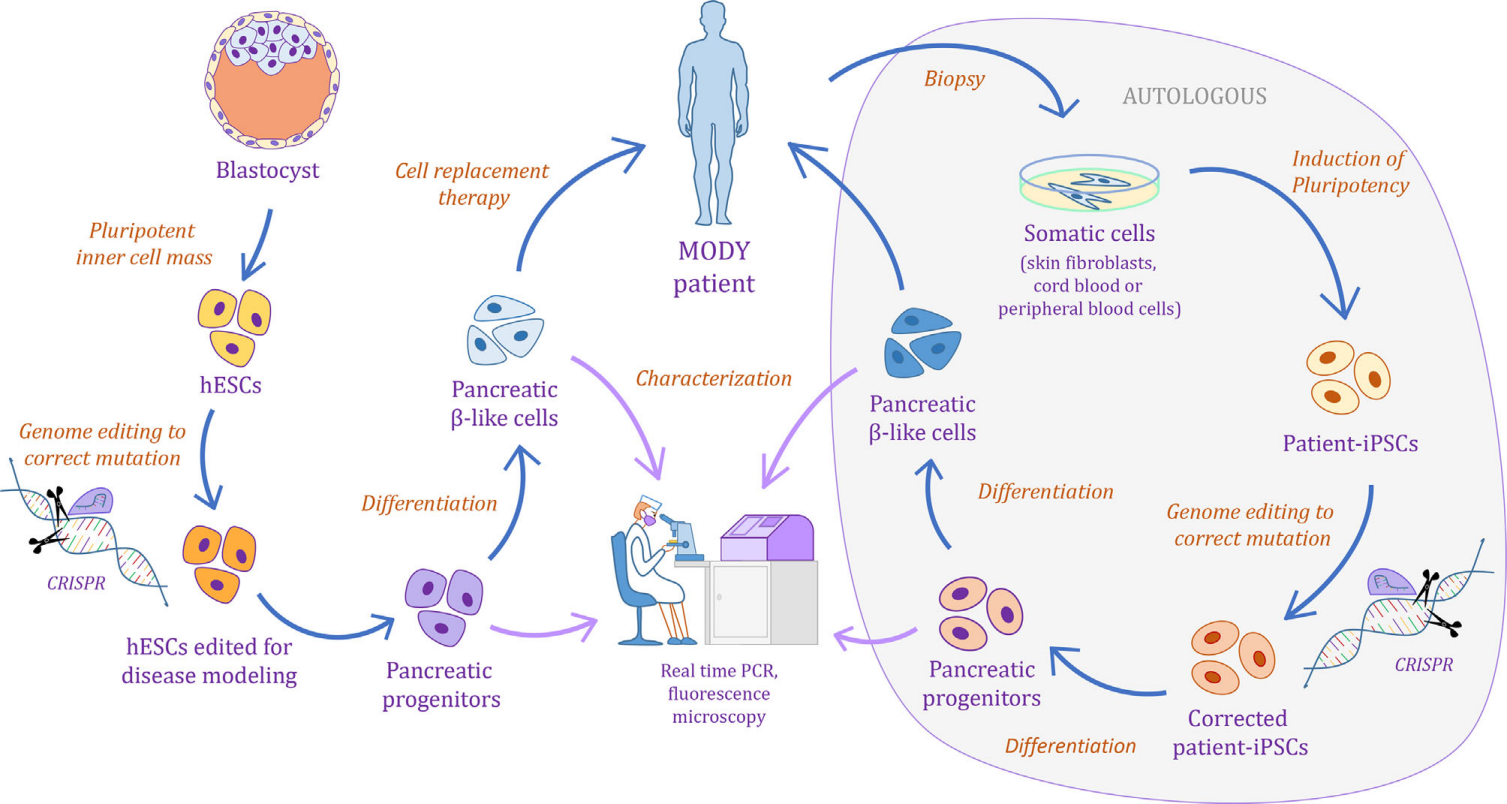
Schematic illustrating the pipeline for monogenic diabetes disease modeling using human pluripotent stem cells.

Monogenic diabetes is caused by either splice-site, non-sense, missense, or frame-shift mutations, and more rarely partial or full deletions, affecting a single gene (8–14). The disease phenotype and associated extra-pancreatic features vary depending on the affected gene (15). These characteristics have made monogenic diabetes an interesting disease subtype to model using human pluripotent stem cells (hPSCs). Indeed, hPSCs can be differentiated into pancreatic cells following key steps of differentiation induced by well-established combinations of growth factors and small molecules, thereby respecting a natural path of development [recently reviewed in (16, 17)]. Thus, differentiation of human induced pluripotent stem cells (hiPSCs) either derived from patients with monogenic diabetes or genetically edited to carry the mutation of interest can be used to study the potential regulatory mechanisms affected by each of the disease-associated genes (**Figure 1**). Applying this approach to a diversity of genes has led to the discovery of new mechanisms associated with specific regulators of pancreatic development. The focus of this review is to discuss the latest findings obtained by modeling monogenic diabetes using hPSC-derived pancreatic cells generated *in vitro*. We will focus on the interpretation of these studies, the advantages and limitations of the models used, and the future perspectives for improvement. Of note, the reader is referred to recent reviews concerning: 1) the state-of-the-art knowledge in pancreatic *β* cell development in mice and humans (18–21); 2) the tools for hPSC genome editing (22, 23); 3) a comparison of the *in vitro* pancreatic differentiation protocols including the latest advances to achieve functional *β* cells from hPSCs (16, 17, 24); 4) analyzing the intrinsic variation in the protocol outcomes from different sources of hPSCs (25, 26); and 5) the use of *in vitro* pancreatic differentiation from hPSCs to discover new mechanisms underlying human pancreas development (24, 27), or to model other types of diabetes (23, 28–31). The later also summarize findings on monogenic diabetes modeling. Our review adds up on top of these by exclusively focusing on the modeling of monogenic diabetes, discussing in more detail the different approaches taken and including extremely recent works which provide insightful information for the interpretation of the results published so far.

## Human Pancreas Development

### Lessons Learned From Mice and Current Challenges

Pancreas development begins with the establishment of the pancreatic bud containing multipotent pancreatic progenitor cells (MPCs) at ~E8.5 in the mouse (19, 20) or ~29 days post conception in humans (20, 21, 32) and progresses until E18.5 in the mouse (19, 20) or 24 weeks post conception (wpc) estimated in humans (20, 21, 33–35). By this time, most of the pancreatic progenitor cells are terminally fate-committed. The MPCs are capable of differentiating along the three main lineages of the adult pancreas, namely the ductal, the exocrine (comprising acinar cells that secrete digestive enzymes), and the endocrine (including the *β* cells that produce insulin, but also the *α*, *δ*, *γ*, and PP cells) (19, 36, 37). As the pancreas develops, MPCs differentiate into acinar or endocrine-ductal bipotent progenitor (BP) cells, and eventually to endocrine-committed progenitors (EPs) that will give rise to *β* cells. Importantly, recent single cell RNA-seq (scRNA-seq) studies have described the transcriptional profiles that characterize these pancreatic progenitor cell stages in the mouse and identified additional progenitor cell sub stages (38–42). Thus, we can now rely on a precise transcriptomic fingerprint for several progenitors that arise during mouse pancreas development.

Cumulative knowledge has revealed the role and stage-specific functions of signaling pathways in the pancreatic developmental program, including Wnt, TGF-*β*, Notch, FGF and, more recently, the Hippo pathway (19, 36, 37, 43–46). The pancreatic mesenchyme has also been shown to play important roles during development, by fine-tuning the crosstalk with the pancreatic epithelium through these pathways (47–50). This knowledge has been exploited to develop protocols to differentiate human embryonic stem cells (hESCs) into the pancreatic lineage (51–55), opening the possibility to produce large quantities of *β* cells for cell-based therapies and also providing a new avenue for research in human pancreas development.

Differentiation protocols currently available to produce *β* cells from hPSCs take advantage of the cell signaling events that occur during fetal development (18–21). Despite this knowledge-based approach, the generation of fully functional *β* cells *in vitro* has remained elusive. We thus refer to the cells produced *in vitro* so far as *β*-like cells. This limitation could be in part explained by the fact that the function of these pathways during pancreas development has been mainly studied in rodents, and it has recently been reported that human pancreas development could differ in several aspects (20, 21, 32, 56, 57). We are still lacking systematic studies comparing islet development between human and mouse, although recent reports are moving forward to address this gap (57–60). It is expected that a deeper understanding of these inter-species differences in islet development will probably be critical for the production of fully functional and mature *β* cells from hPSCs. As well, this will allow a more precise dissection of the molecular mechanisms driving diabetes predisposition by genetic mutations and/or external stimuli. Noteworthy, recent protocols allow the derivation of monohormonal insulin-producing cells expressing key *β* cell transcription factors, including *PDX1*, *NKX6.1*, and *MAFA* (61–65). Fine tuning of such protocols could further improve the glucose-response of *in vitro* derived *β*-like cells (66–69) without the need to involve a step of cell transplantation in mice to ensure proper maturation.

### Lessons Learned From *In Vitro* Human PSC Pancreatic Differentiation

The use of hPSCs as *in vitro* model system to study human pancreatic development has gained momentum and several important discoveries have been made using this approach [recently reviewed in (27)].

#### A Role for TEAD and YAP in Pancreas Development

By comparing the transcriptomes and key epigenomic features of MPCs derived *in vitro* from hESCs with human fetal primary pancreatic tissue of six wpc embryos, we were able to show that *in vitro* derived cells closely recapitulated the main expression profile and regulatory landscape of their *in vivo* counterparts (45). Furthermore, this epigenomic characterization was extended to include ChIP-seq profiling of several transcription factors by taking advantage of the *in vitro* system. A combined analysis of these data led to the discovery that TEAD1 was an integral component of the enhancer network in human embryonic pancreatic progenitors (45, 46). The relevance of TEAD protein binding for the activation of MPC enhancers was mechanistically validated using the platform provided by hPSC-derived pancreatic cells *in vitro*. We concluded that, while highly tissue specific enhancers were defined by co-binding of pancreas-specific transcription factors, TEAD proteins conferred these regions the ability to be regulated by YAP (an effector of the Hippo pathway) during human pancreas development. These results were consistent with reports for a role of the Hippo pathway in mouse pancreas development (43, 44). More recent reports support these findings by showing that YAP and other components of the Hippo pathway are active and highly enriched within the SOX9+/PTF1A+ progenitor cells of the human fetal pancreas (58).

The human *in vitro* differentiation system has provided additional mechanistic evidence for the relevance of the Hippo pathway in pancreas differentiation by showing that YAP links extracellular matrix-mediated mechano-signals to regulate gene expression. Integration of such signaling plays a key role in the fate choice of bipotent pancreatic progenitors, whereby YAP downregulation favors endocrine cell commitment (70). In agreement, sustained *in vitro* YAP activation impairs *β* cell differentiation while inhibition of YAP enhances differentiation of functional *β* cells derived from hPSCs (71). Combined together, these results illustrate how *in vitro* pancreatic differentiation can help in the discovery of new regulatory mechanisms that are relevant for human *β* cell development.

#### A Role for Polycomb Group-Mediated Repression in Pancreas Development

Mapping the dynamic changes in histone modifications and chromatin accessibility across different stages of the *in vitro* pancreatic differentiation protocol has provided important insights into the suitability and current limitations of this model (72–75). It has to be noted that, despite successful applications, the *in vitro* pancreatic differentiation system does not exactly replicate all the epigenomic features of their *in vivo* counterparts. Sander and colleagues have profiled selected chromatin modifications and the transcriptome of these cells, at different stages of the pancreatic endocrine differentiation protocol using hESCs (72). They showed that removal of Polycomb group (PcG)-mediated repression on stage-specific genes was a key mechanism for the induction of developmental regulators in the *in vitro* system, consistent with the *in vivo* relevance of this mechanism in mouse endocrine pancreas development (76–78). However, they also reported that elimination of PcG-mediated repression on endocrine-specific genes was not fully recapitulated by the *in vitro* derived endocrine cells. This was particularly evident at genes involved in organ morphogenesis, underscoring a current limitation (*i.e.* the lack of tissue-specific contextual cell signaling) of the *in vitro* protocols for studying some aspects of *in vivo* development. Noteworthy, these experiments were performed using 2D *in vitro* differentiation protocols which have been shown to be less efficient to produce functional *β*-like cells than three-dimensional suspension culture systems (61–63, 66–68). This “second generation” protocols could be more efficient in allowing the proper deposition of epigenetic marks.

#### Distinct Progenitor Cell Populations Could Differentiate Into Monohormonal *β* Cells

Another example of the complexity of the *in vitro* endocrine differentiation process was provided by Petersen et al. who profiled single-cell transcripts by qRT-PCR at selected stages of the protocol used to produce *β*-like cells from hPSCs (79). This analysis identified two distinct progenitor cell populations with the potential to differentiate into monohormonal *β*-like cells. *NKX6.1* expression prior or after the onset of *NEUROG3* (the gene coding for the EP transcription factor marker NGN3) was the main difference between these progenitors. Building up on these results, Ramond et al. performed a combined analysis of single-cell qRT-PCR datasets obtained from pancreatic progenitor and endocrine cells from *in vitro* and *in vivo* samples (59, 60). Their observations suggest that these distinct progenitor cell populations identified *in vitro* could indeed exist during *in vivo* development. This finding contrasts with knowledge gained from mouse studies where Nkx6 factors systematically specify endocrine cell fate upstream of Ngn3 in the MPC stage (80, 81). Still, the function and characteristics of these two populations of progenitors in the developing human pancreas remain to be fully elucidated.

Taking advantage of novel single-cell technologies can help to match some of the transcriptional signatures from *in vivo* pancreatic progenitor cell stages identified in the mouse (38–41), with pancreatic progenitors derived from hPSCs (38, 66, 82). However, such exercise remains challenging due to inter-species differences and also the impact of the *in vitro* culture. New tools are being quickly developed to address this limitation, making the bioinformatic analyses of these data an exciting area of research (83, 84). More recently, the first scRNA-seq experiments using human embryonic pancreas from 15.2 and 17.1 wpc have been reported (58, 85). Integration of these scRNA-seq datasets with those derived from the mouse embryonic pancreas will help to identify differences and similarities in the transcriptional fingerprints of the distinct pancreatic progenitor cell types. This will ultimately contribute to validate the identity of pancreatic progenitors produced from hPSCs.

## Modeling Monogenic Diabetes With Human Pluripotent Stem Cells

The most frequently affected maturity-onset diabetes of the young (MODY) genes include the enzyme glucokinase (*GCK*, MODY2) (86, 87) and the transcription factor genes hepatic nuclear factor 1 alpha (*HNF1A*, MODY3) (88), hepatic nuclear factor 4 alpha (*HNF4A*, MODY1) (89), and hepatic nuclear factor 1 beta (*HNF1B*, MODY5) (90). Other MODY genes include *PDX1* (MODY4), *NEUROD1* (MODY6), *KLF11* (MODY7), *CEL* (MODY8), *PAX4* (MODY9), *INS* (MODY10), *BLK* (MODY11), *ABCC8* (MODY12), *KCNJ11* (MODY13), *APPL1* (MODY14) (91). On the other hand, homozygous mutations at several lineage determining transcription factors, such as *PTF1A*, *PDX1*, *NEUROG3*, *RFX6*, *NEUROD1*, *MNX1*, *NKX2.2* and *GLIS3* result in permanent neonatal diabetes mellitus (PNDM) in humans (13, 92–100). Interestingly, heterozygous mutations in these genes rarely result in diabetes in mice, thereby suggesting an important divergence in the activity or function for these factors between human and mouse (19, 21, 101). The importance of haploinsufficiency and the mechanisms by which a decrease in transcription factor activity causes a disease in humans is poorly understood, mostly due to the lack of an appropriate model system. As an example, MODY5 diabetes (*HNF1B*-associated) can be induced by a diversity of mutations including several splice-site, non-sense, missense, and frame-shift mutations or whole gene deletions, all of which result in a diabetes (102). The heterozygous mutation in mouse has no effect on pancreatic development, while homozygous mutation blocks foregut specification thereby masking its downstream function in the differentiation of MPCs. Ultimately, haploinsufficiency may reflect the functional effects of different gene anomalies, stochastic variation in temporal gene expression during early development or additional genetic and/or environmental modifiers that may influence the disease phenotype (102–104).

As mentioned above, mouse models often do not recapitulate the disease phenotype associated with heterozygous mutations of *HNF1A*, *HNF4A*, or *HNF1B* in humans. The genetic discrepancy between the mouse and monogenic diabetes gene haploinsufficient patients and the difficulty in accessing patient samples have reinforced the interest in using hPSCs (**Figure 1**). Genome-editing tools combined with directed differentiation of hPSCs offer a unique platform for generating patient-specific disease models to elucidate novel genes and molecular pathways that underlie monogenic diseases with complex traits, such as diabetes, and ultimately lead to the development of novel therapeutic strategies [recently reviewed in (22, 25)]. Several studies in the last decade have used genetically engineered hPSC culture systems for differentiation into pancreatic cells to further expand our understanding of the roles of various genes associated with monogenic diabetes. Their findings are summarized in **Tables 1**, **2**, and these will be discussed in more detail next.

**Table 1:** Summary of reports modeling maturity-onset diabetes of the young (MODY) mutation effects.

Gene studied	Pancreatic defects reported in humans	Effects recapitulated in mice	Genome editing approach	Differentiation protocol	Type of human pluripotent stem cell	*In vitro* phenotypes	Ref.
***HNF4A* (MODY1)**	*HNF4A* heterozygous mutations affect both liver and pancreas development. MODY1 patients present neonatal hyperinsulinemia and impairment in β cell function. They present normal insulin sensitivity but decreased insulin secretion.	Rodent models do not accurately recapitulate the MODY1 phenotype in humans. The available *Hnf4a* general knockout murine model is embryonic lethal, while heterozygous mice present normal glucose tolerance and do not show any diabetic features.	NA	(62)	hiPSCs were derived from MODY1 mutation carriers. Their family members, without the mutation, were used as controls.	The *HNF4A* mutation studied did not prevent the formation of insulin+ cells *in vitro*. Also, no defects in *β*-like cells differentiated from *HNF4A* mutant hiPSCs were found.	(105)
			NA	Adapted from (62).	Control hiPSC lines (CSES7 and IPSO lines) and MODY1 patient-derived hiPSCs.	Researchers report that cells from the MPC stage show increased expression of endocrine progenitor transcription factors, including *PAX6*, *NEUROD1* and *NEUROG3*.	(106)
			Site-directed mutagenesis.	(61)	hiPSCs were derived from non-diabetic and MODY1 patients.	Key developmental genes such as *HNF1B*, *PDX1*, *GATA4*, and *RFX6* are downregulated at the foregut progenitor stage, prior to MPC specification. Still, terminally differentiated β-like cells can be produced and express selective β cell markers and C-peptide. The functional capacity of these cells could not be appropriately elucidated due to limitations of the *in vitro* protocol used.	(107)
***GCK* (MODY2)**	Patients with *GCK* heterozygous mutations present progressive β-cell dysfunction, fasting hyperglycemia and reduced insulin secretion. These result in a mild diabetes phenotype that generally does not require anti-diabetes medication.	Homozygous mutant mice exhibit growth retardation and die soon after birth as consequence of severe hyperglycemia. Heterozygous mutant mice only present slightly elevated blood glucose levels from birth, with disturbed glucose tolerance and glucose-induced insulin secretion.	NA	NA	Non-edited MODY2 and PNDM patient-derived hiPSCs.	This work reports the generation of iPSCs from MODY2 patients. The researchers did not analyze differentiation into the pancreatic lineage.	(108)
**HNF1A (MODY3)**	Patients with *HNF1A* heterozygous mutations show β cell dysfunction and hyperglycemia due to insufficient insulin release in response to increased blood glucose levels.	Mouse models do not fully mimic the human disease phenotype. Mice with heterozygous mutations in *Hnf1a* are healthy and mice with homozygous null mutations present a diabetic phenotype.	CRISPR-CAS9 system.	(62), with minor modifications.	Genome-edited hESCs (MEL1 and H1) and human β-cell lines (EndoC-BH).	Differentiation from *HNF1A* ^+/-^ hESC show reduced number of INS+ cells. β-like cells present defects in mitochondrial function and the glycolysis process. Decreased expression of β cell transcription factors and genes associated with insulin synthesis. Reduced β cell proliferation and increased apoptosis.	(109)
			NA	(61), with some modifications.	hiPSCs were derived from MODY3 patients. hiPSCs derived from a healthy donor were used as control.	*HNF1A* MODY3 mutations caused decreased *GLUT2* expression, which was associated with reduced glucose uptake and ATP production. The mutant *HNF1A* β-like cells present decreased insulin secretion in response to high glucose.	(110)
***PDX1* (MODY4)**	*PDX1* heterozygous mutations are associated with insulin secretion deficiency. Common point heterozygous mutations in the *PDX1* transactivation domain impair human pancreatic β cell formation and function, and contribute to increased risk for diabetes. Pancreatic developmental anomalies related to *PDX1* mutations are reported only in neonatal diabetes cases.	Homozygous *Pdx1*-deficient mice fail to generate a pancreas, while heterozygous animals develop a pancreas but become diabetic in adulthood due to β cell apoptosis.	TALEN and CRISPR/Cas9.	Adapted from (52, 54).	Genome-edited hESCs (HUES8).	Monoallelic *PDX1* mutations are associated with decreased PDX1 protein expression. These compromise endocrine differentiation and lead to reduction in the number of INS+ cells derived *in vitro.*	(111)
			CRISPR/Cas9.	Based on (62).	Genome-edited hiPSCs and patient-derived hiPSCs.	Heterozygous mutations impair *in vitro β* cell differentiation and function. Homozygous point mutations in the *PDX1* transactivation domain do not only impact pancreatic endocrine lineage development, but also impair glucose-responsive function of β cells through misregulation of several PDX1 target genes.	(112)
***HNF1B* (MODY5)**	Patients with *HNF1B* heterozygous mutations commonly exhibit pancreatic hypoplasia, β-cell dysfunction and insulin resistance.	*Hnf1b^-/-^* mice present pancreatic agenesis, exhibiting loss of expression of several pancreatic genes, including Pax6, which regulate β-cell function. In contrast with MODY5 patients, *Hnf1b* ^+/-^ mice do not develop diabetes.	NA	Adapted from (52).	MODY5 patient-derived hiPSCs.	Upregulation of multiple key pancreatic transcription factors at the DE and MPC stage, including *FOXA2*, *PDX1*, *GATA4* and *GATA6*. Interestingly, expression of HNF1B itself was induced in mutant hiPSC-derived MPCs. Reduction of PAX6 expression.	(113)

NA, not applicable.

**Table 2:** Summary of reports modeling monogenic mutations associated with permanent neonatal diabetes mellitus (PNDM) or pancreatic agenesis.

Gene studied	Pancreatic defects reported in humans	Effects recapitulated in mice	Genome editing approach	Differentiation protocol	Type of human pluripotent stem cell	*In vitro* phenotypes	Ref.
***GATA6***	*GATA6* heterozygous inactivating mutations result in pancreatic agenesis.	*Gata6* heterozygous mice are fertile and phenotypically normal. *Gata6* null mice are embryonic lethal. Biallelic loss of *Gata6* and its paralog *Gata4* result in a phenotype similar to human PNDM *GATA6*-mutated patients.	CRISPR/Cas9-mediated genome editing.	Adapted from (52).	Patient-derived hiPSCs and genome-edited hESCs. Isogenic, mutation-corrected, hiPSCs were used as controls.	*GATA6* homozygous mutations lead to impaired DE differentiation. Rescue of DE defects in these cells by re-expression of other GATA family members allows β-like cell production with a lower efficiency. hPSCs with *GATA6* heterozygous mutations show defects in DE differentiation. β-like cells produced in both cases are defective in the GSIS and in insulin processing.	(114)
			CRISPR/Cas9-mediated genome editing.	(61, 62, 111), with some modifications.	Genome-edited hESCs (H1 and HUES8).	Differentiation of *GATA6* ^-/-^ hPSCs revealed impaired DE commitment and pancreatic endocrine differentiation. No defects in DE differentiation from *GATA6* ^+/-^ hPSCs, but a lower number of *PDX1+ NKX6.1+* pancreatic progenitors and β-like cells was produced.	(115)
			TALENs	(55), adapted from (52).	hiPSCs derived from pancreatic agenesis patients with *GATA6* heterozygous mutations. Genome-edited hESCs (H9) and hiPSCs. Non-mutated hESCs and hiPSCs were used as isogenic controls.	*GATA6* heterozygous hPSCs present a modest decrease in the generation of DE, which differentiate less efficiently into MPCs and EPs. *GATA6*-null hPSCs fail to enter the DE lineage.	(116)
			CRISPR-CAS9-mediated genome editing.	Adapted from (61, 62, 54).	hiPSCs derived from a patient with pancreatic agenesis. Isogenic, mutation-corrected hiPSCs were used as control.	hiPSCs with *GATA6* heterozygous mutations present reduced efficiency for generation of pancreatic progenitor cells *in vitro*. Correction of these mutations allowed identifying a non-coding SNP that additionally contributes to the phenotype observed.	(117)
***PDX1***	Homozygous mutations in *PDX1* result in pancreatic agenesis. PDX1 heterozygous patients exhibit diabetes caused by defects in β cell function and/or the maintenance of β cell mass in adults.	Homozygous mutations in *Pdx1*cause pancreatic agenesis, while heterozygous animals develop a pancreas but become diabetic in adulthood due to β cell apoptosis.	TALEN ad CRISPR/Cas9.	Adapted from (52, 54).	Genome-edited hESCs (HUES8).	Differentiation of *PDX1* ^+/-^ mutant hESCs present a 65% reduction of INS+ cells at the β-like cell stage, which are mainly polyhormonal cells using the protocol described in this study.	(111)
***RFX6***	Patients carrying biallelic *RFX6* inactivating mutations present a reduction in the pancreas size and obstruction of the small intestine. These patients present defects in the formation of pancreatic progenitors and their further differentiation into functional endocrine cells.	Similar to humans, *Rfx6*-null mice show variable degrees of pancreatic hypoplasia and premature death.	TALEN ad CRISPR/Cas9.	Adapted from (52, 54).	Genome-edited hESCs (HUES8).	Differentiation of *RFX6* ^-/-^ mutant hESCs show a reduction in the number of *PDX1+* pancreatic progenitor cells. Severe reduction in β-like cells and complete absence of α cells.	(111)
			CRISPR/Cas9-mediated genome editing.	Adapted from (62)	hiPSCs were derived from patients with MRS and from their healthy, heterozygous father. hESCs (H9) was used as control.	hiPSCs with *RFX6* homozygous mutations show normal DE and PFG differentiation, but fail to robustly activate *PDX1*. MPCs and endocrine-competent progenitors differentiate less efficiently from these cells.	(118)
***PTF1A***	Homozygous inactivating mutations in *PTF1A* cause pancreatic and cerebellar agenesis.	*Ptf1a-*null mice present a complete absence of exocrine pancreatic tissue, but all islet endocrine cell types are present until the late stages of embryogenesis.	TALEN ad CRISPR/Cas9.	Adapted from (52, 54).	Genome-edited hESCs (HUES8).	Differentiation of *PTF1A* ^-/-^ mutant hESCs do not present defects in pancreatic endocrine differentiation using the protocol described.	(111)
***GLIS3***	Biallelic mutations of *GLIS3* underlie a rare clinical syndrome, characterized by neonatal diabetes and congenital hypothyroidism.	Global *Glis3* ^-/-^ mice die of severe neonatal diabetes shortly after birth. Minor differences in gene dosage of Glis3 produce substantive changes in the expression levels of Ngn3 and Ins1, leading to a variable phenotype among the multiple *Glis3*-KO mouse lines.	TALEN ad CRISPR/Cas9.	Adapted from (52, 54).	Genome-edited hESCs (HUES8).	Differentiation of *GLIS3* ^-/-^ mutant hESCs do not present defects in pancreatic endocrine differentiation using the protocol described.	(111)
			CRISPR/Cas9-mediated genome editing.	(64)	Genome-edited hESCs.	Differentiation of *GLIS3* ^-/-^ mutant hESCs show impaired expression of pancreatic endocrine-associated genes, including *PDX1*, *NEUROD1*, *NKX6.1*, and *MAFA*, and present increased β-like cell death. A chemical screen identified a drug candidate that rescues mutant GLIS3-associated β-cell death both *in vitro* and *in vivo*.	(64)
***MNX1***	Homozygous mutations in *MNX1* are associated with the occurrence of diabetes in infancy without evidence of exocrine pancreatic dysfunction. Reduced number of pancreatic endocrine cells, including β cells.	*Mnx1*-deficient mice show pancreatic dorsal-lobe agenesis and smaller pancreatic islets, while *Mnx1* gain-of-function in the pancreas leads to aberrant pancreatic development.	TALEN ad CRISPR/Cas9.	Adapted from (52, 54).	Genome-edited hESCs (HUES8).	Differentiation of *MNX1* ^−/−^ mutant hESCs do not present defects in pancreatic endocrine differentiation using the protocol described.	(111)

DE, definitive endoderm; MPC, multipotent pancreatic progenitor cells; PFG, posterior foregut; GSIS, glucose-stimulated insulin secretion; MRS, Mitchell-Riley syndrome; KO, knock out; ER, endoplasmic reticulum.

### WFS1

Egli and colleagues provided the first example for the use of hiPSCs to create insulin-producing cells from patients with Wolfram Syndrome (WS) (119). hiPSCs were generated from individuals with diabetes caused by mutations in the *WFS1* gene and healthy-donor controls. Differentiation of these cells towards *β*-like cells revealed increased levels of ER stress molecules and decreased insulin content in WFS1-deficient *β*-like cells. Overall, insulin processing and secretion in response to various secretagogues was comparable to healthy controls, but the former displayed increased activity of unfolded protein response (UPR) pathways.

More recently, Maxwell et al. used CRISPR/Cas9 to correct a diabetes-causing pathogenic variant in *WFS1* hiPSCs (120). Noteworthy, *β*-like cells differentiated from *WFS1*-corrected hiPSCs showed robust and dynamic insulin secretion in response to glucose, and reversed streptozocin-induced diabetes when transplanted into mice. Single-cell RNA-seq transcriptome profiling showed that indeed these cells displayed increased insulin levels and decreased expression of genes associated with endoplasmic reticulum stress. Taken together, these studies illustrate the potential of *in vitro* pancreatic differentiation from hPSCs to study how mechanisms related to cellular stress can affect diabetes onset.

### PDX1

Homozygous null mutations in *PDX1* result in pancreatic agenesis both in mice and humans (13, 121–123). Human patients with *PDX1* heterozygous inactivating mutations exhibit MODY4 diabetes caused by defects in *β* cell function and/or the maintenance of *β* cell mass in adults (36). In rodents, it has been reported that *Pdx1* ^+/−^ mice can develop a functional pancreas (121, 122) but become diabetic in adulthood due to *β* cell apoptosis (124).

Another pioneer study to model monogenic diabetes was reported by Huangfu and colleagues, who used TALEN and CRISPR-Cas-mediated gene editing combined with hPSC-directed differentiation. These researchers provide a systematic analysis of the role for PDX1 and seven additional pancreatic transcription factors (RFX6, PTF1A, GLIS3, MNX1, NGN3, HES1 and ARX) in pancreatic cell commitment (111). Noteworthy, they created mono- or biallelic frameshift mutations in all these genes and used untargeted isogenic cell lines as controls. This analysis not only defined the specific developmental steps affected by these mutations in a model of human pancreas differentiation, but also revealed new mechanisms. **Tables 1**, **2** show a summary of their results for the genes previously associated with MODY and/or PNDM. An interesting finding of this work was that monoallelic frameshift translation mutations disrupting the PDX1 protein sequence cause a reduction (up to 65%) in the number of insulin+ cells derived *in vitro*. These findings suggest a haploinsufficient requirement for *PDX1* in pancreatic endocrine development. Importantly, this phenotype correlates with the observation that patients with heterozygous mutations in *PDX1* present with diabetes from an early age (125). These results further validate that decreased amounts of PDX1 could lead to *β* cell dysfunction, a decrease in *β* cell mass during fetal development and/or the maintenance of *β* cell mass in adults (124, 126, 127).

More recently, Lickert and colleagues generated hiPSCs from two patients with heterozygous missense mutations in the *PDX1* coding region (*PDX1^P33T/+^* and *PDX1^C18R/+^*) leading to single amino acid exchanges in its transactivation domain (112). By comparing with a control hiPSC line derived from a healthy donor, the authors showed that MPC differentiation was not affected in patient-derived hiPSCs. However, the *PDX1* heterozygous point mutations impaired the differentiation of *β*-like cells and affected their response to glucose. A more severe effect was observed when artificially introducing the same point mutations in homozygosis (*i.e. PDX1^P33T/P33T^* and *PDX1^C18R/C18R^*) in isogenic cell lines derived from the original control cell. Interestingly, this resulted in impaired *NKX6.1* induction in MPCs just in one of the cell lines (*PDX1^P33T/P33T^*). Nevertheless, when differentiated towards insulin producing cells, both homozygous cell lines yielded a decreased number of *β*-like cells with impaired glucose response. The authors also generated additional isogenic lines carrying different heterozygous mutations in the *PDX1* transactivation domain, to generate a frame-shift mutation (*PDX1^+/^* ^−^). This created a more severe phenotype to the one observed in the patient-derived hiPSCs, leading to similar outcomes as obtained from the homozygous isogenic *PDX1^P33T/P33T^* point mutated cells. Further transcriptomic analyses of MPCs differentiated from these cell lines ascribed the observed effects to downregulation of key PDX1-bound genes including *MEG3* and *NNA*, which are involved in pancreas development and insulin secretion.

Taken together, these results illustrate how predisposition to develop diabetes can be provoked at the stage of pancreatic endocrine lineage development by genetic mutations on a gene that plays a key role at this timepoint. These anomalies could impair the glucose-responsive function of *β*-like cells through misregulation of genes involved in *β* cell development, maturation, and function. These results also emphasize that the choice between patient-derived hiPSCs or healthy donor hiPSCs with mutations artificially introduced, as well as the choice of the control cell line used, can affect experimental outcomes and their interpretations. In this context, patient-derived hiPSCs could carry additional mutations in non-coding regulatory regions and/or other genes which might further impair the *in vitro* differentiation outcomes. This effects have been elegantly exposed in a recent work by Gadue and colleagues (117), which will be discussed in more detail below, in the *GATA6* section of this review. In contrast, the use of healthy donor hiPSCs with mutations artificially introduced has the advantage of enabling the use of isogenic cell lines (*i.e.* non-mutated hiPSCs) to exclude additional effects of the genetic background.

### RFX6

Lack of Rfx6 in mice blocks differentiation of all islet cell types, with the exception of pancreatic-polypeptide-producing cells, while *RFX6* mutations in humans result in PNDM (93, 100, 128). Modeling of the RFX6 requirement for human endocrine pancreas development has been addressed by Zhu et al. Their findings, in agreement with current knowledge, show a reduction of endocrine cell commitment from pancreatic progenitor cells derived from *RFX6* ^−^ */* ^−^ mutant hPSCs (111).

In a more recent study, Trott et al. used hiPSCs derived from individuals with Mitchell–Riley syndrome (MRS) to specifically associate the role of *RFX6* mutations and the lack of pancreatic endocrine cells in a human model of pancreas development (118). X-ray microtomography of one of these patients confirmed the spectrum of congenital defects typical of MRS (loss of the pancreas body and tail), and exome sequencing identified a homozygous non-sense mutation in *RFX6*. hiPSCs derived from this patient and differentiated along the pancreatic cell lineage revealed that these cells efficiently differentiate into posterior foregut cells but exhibited a reduction in the pancreatic endoderm differentiation, which was accompanied by expression of genes associated with mesoderm differentiation. These findings indicate that RFX6 is crucial for maintaining the transcriptional program that specifies early pancreatic endoderm in humans.

### NEUROG3

While loss of Ngn3 function has been associated with complete lack of pancreatic endocrine cells in mice (129), the phenotype in humans is variable [recently reviewed in (21)]. In this sense, while some patients with homozygous or compound heterozygous *NEUROG3* mutations show glycemic control into adulthood, indicating a functional endocrine pancreas, others present neonatal diabetes (96, 130, 131). A recent study suggests that each mutation could have unique effects on the structure and function of NGN3 (132). To further understand this divergence, the requirement of NGN3 for the generation of insulin-producing cells during human development has been addressed using hPSC differentiation. Zhu et al. reported that *in vitro* endocrine pancreatic differentiation of hPSCs with biallelic mutations in *NEUROG3* formed some insulin-producing cells (111), whereas another study reported a total lack of endocrine cells differentiated from *NEUROG3* ^−/−^ hPSCs (133). The latter work described that as little as 10% *NEUROG3* expression is sufficient for the formation of pancreatic endocrine cells, supporting that NGN3 is essential for endocrine pancreas development in humans. The divergence between differentiation protocols used in each laboratory and the influence of genetic background could explain the varied phenotypes observed between these two studies. Interestingly, a new adult mouse islet resident pancreatic endocrine progenitor cell population has been recently reported (134). These cells express the surface marker *Procr*, are *Neurog3* negative and, when isolated and co-cultured with endothelial cells, are able to give rise to islet-like clusters containing all endocrine cell types. Apparently, differentiation of this adult progenitor cell population into endocrine cells does not involve *Neurog3* expression, raising the intriguing question of whether such a population exists in humans and, if so, whether *in vitro* pancreatic differentiation from hPSCs is able to follow this “alternative” path for endocrine cell production. Such possibility could explain the divergence between different reports concerning the requirement of NGN3 in endocrine cell production. Taken together, these studies illustrate the complexity, as well as the potential, associated with hPSC differentiation for modeling the impact of genetic mutations on human development.

### GLIS3

It has been reported that global *Glis3* ^−/−^ mice die of severe neonatal diabetes shortly after birth (135). Minor differences in gene dosage of *Glis3* produce substantive changes in the expression levels of *Neurog3* and *Ins1*, leading to a variable phenotype among the multiple *Glis3*-KO mouse lines (136). In agreement with these phenotypes, human biallelic mutations in *GLIS3* underlie a rare clinical syndrome, characterized by neonatal diabetes and congenital hypothyroidism (92).

The first report of the *in vitro* modeling for the requirement of GLIS3 in human pancreas development was provided by Zhu et al. These researchers did not find defects in pancreatic endocrine differentiation using *GLIS3* ^-/-^ mutant hESCs, when using a first generation *in vitro* pancreatic differentiation protocol that allows producing poly-hormonal cells (111).

More recently, Amin et al. developed an improved differentiation protocol that allowed the production of monohormonal *β*-like cells with enhanced functionality (64). Noteworthy, this protocol allowed the generation of robust *GLIS3* expression at the PDX1+/NKX6.1+ pancreatic progenitor cell stage, in contrast with previously reported protocols (52, 111). Using this improved protocol, they were able to demonstrate that differentiation of *GLIS3* ^−/−^ mutant hESCs presented impaired expression of pancreatic endocrine-associated genes, including *PDX1*, *NEUROD1*, *NKX6.1*, and *MAFA*. These cells also showed increased *β*-like cell death. These findings contrast with those reported by Zhu et al. (111). The difference could be explained by the improvements in the differentiation protocol, which allow a closer recapitulation of the differentiation steps to produce *β*-like cells. Furthermore, providing an illustrative example of the utility of the *in vitro β* cell differentiation protocols, these researchers performed a chemical screen that allowed the identification of a novel drug candidate that rescued mutant *GLIS3*-associated *β*-cell death both *in vitro* and *in vivo* (64).

### HNF1A

Hnf1a has been shown to regulate the expression pattern of islet-specific genes involved in key functions of this tissue (137). In the mouse, while homozygous knockout (*Hnf1a* ^−/−^) results in insulin secretory defects and higher blood glucose concentrations, heterozygous knockout (*Hnf1a* ^+/−^) do not display this phenotype (138). This is in sharp contrast with the MODY3 pathology in humans, in which heterozygous mutations result in diabetes (139). In an attempt to elucidate the mechanisms by which dysfunctional HNF1A affects pancreatic development and/or *β* cell function, Gadue and colleagues have modeled MODY3 using CRISPR-Cas9 genome-edited hESCs and EndoC-BH human cell lines (109). Loss of HNF1A function was accomplished by deletion and premature termination in one or both *HNF1A* alleles, resulting in heterozygous and homozygous KO mutations. Their results suggest that HNF1A plays an essential role in endocrine cell development, as its loss leads to abnormal expression of genes related to *β* cell function and diabetes. Noteworthy, complete loss of HNF1A did not impair the production of pancreatic progenitors, but this factor was necessary for proper endocrine cell development as revealed by decreased expression of *PAX4*, and impaired insulin expression and secretion. Interestingly, HNF1A loss of function (deletion in one or both alleles of *HNF1A*) led to increased expression of *α* cell markers, including glucagon. The authors suggest that the increase found in *α* cells derived from this model system appears to be human-specific, since *Hnf1a* knockout mice do not display this phenotype.

Another key finding of this work was the identification of a previously unannotated human-specific long intergenic non-coding RNA (*lncRNA*). The *LINC01139*, designated *LINKA*, was shown to act as a downstream target of HNF1A. *In vitro* endocrine pancreatic differentiation of *LINKA*-deficient hESCs showed no effect on the production of pancreatic progenitors, but revealed a limited bias towards the production of *α* cells. Furthermore, *β*-like cells produced from *LINKA*-deficient hESCs showed a decrease in maximal respiration capacity to a similar extent as seen in the *HNF1A* heterozygous cells. Taken together, their findings point to a role for *LINKA* in the regulation of a subset of HNF1A target genes with implications in cellular respiration. The *in vivo* relevance of *LINKA* for diabetes onset remains to be explored. Of note, a significant variability was observed in the expression changes among the hESC lines used in this study. These could be partially explained by the impact of the genetic background, which could lead to differences in the efficiency of differentiation protocol when applied to each cell line.

A more recent report was provided by Teo and colleagues (110). These researchers used MODY3 patient-derived hiPSCs to study the impact of a recently reported patient-specific heterozygous HNF1A^+/H126D^ mutation (140). The authors used hiPSCs reprogrammed from a healthy donor and H9 hESCs as two independent wild type controls. Molecular dynamics simulations predicted that the H126D mutation could compromise DNA binding and gene target transcription. Indeed, RNA-seq and ChIP-seq analyses performed on MODY3 hiPSC-derived endocrine progenitors revealed that the expression of several HNF1A gene targets was affected by the mutation. An in-depth analysis of the effects on the *β*-like cells derived from HNF1A^+/H126D^ hiPSCs demonstrated that the *HNF1A* mutation causes a GLUT2 deficiency, that is associated with reduced glucose uptake and ATP production. Their findings reveal the importance of HNF1A in regulating GLUT2 and several genes involved in the MODY3 pathology that may partly account for the lack of insulin secretion clinically observed in these patients. This report extends the findings reported by Cardenas-Diaz et al. (109) by revealing additional mechanisms triggered by the *HNF1A* mutations on the rest of the stimulus-secretion coupling pathway and on HNF1A transcriptional targets in human *β*-like cells. Noteworthy, Teo and colleagues performed RNA-seq and ChIP-seq at the endocrine progenitor cell stage. They did not found a differential regulation of the *LINC01139* (*LINKA*) at this stage, and unfortunately the expression of this lncRNA in *β*-like cells derived from HNF1A^+/H126D^ hiPSCs is not reported. It remains to be elucidated whether *LINC01139* is also downregulated in the latter model. Potential discrepancies on the regulation of this lncRNA could be accounted by the different approaches followed in each work to evaluate the effects of HNF1A haploinsufficiency. On one hand Cardenas-Diaz et al. artificially introduced KO mutations by generating a genomic deletion leading to premature termination in one or both *HNF1A* alleles, and non-mutated isogenic cell lines were used as controls. This approach has the advantage of using an isogenic control cell line, which neutralizes contributions from the genomic background. However, the mutations introduced generate a strong HNF1A loss of function that might not appropriately recapitulate the mechanisms that take place in MODY3 patients. On the other hand, Teo and colleagues used MODY3 patient-derived hiPSCs carrying a mutation that causes an amino acid substitution (HNF1A^+/H126D^) and used hiPSCs derived from a healthy donor and H9 hESCs as wild type controls. This approach has the advantage of using hiPSCs derived from patient cells, accounting for a closer model to the MODY3 disease. However, the use of non-isogenic hPSCs as controls does not allow accounting for potential effects derived from the different genomic backgrounds. As presented in more detail in the next section, these might introduce an additional bias in the differentiation outcome. In summary, further studies are required to elucidate whether deregulation of *LINC01139* plays a relevant role in MODY3 diabetes.

### GATA 6

Mono allelic mutations in *GATA6* have been linked with pancreas agenesis in humans (141) while the knockout of the same gene has little effect on pancreatic development in the mouse. Indeed, only knockout of both *Gata4* and *Gata6* results in pancreatic agenesis (142, 143). Thus, GATA6 seems to have a different or at least a more extensive function in human development. To confirm this observation, Shi et al. used CRISPR/Cas9 to create hPSCs carrying frameshift mutations in *GATA6*, alone or in combination with *GATA4* mutations (115). Their results show that GATA6^+/−^ haploinsufficiency alters pancreatic progenitor cell differentiation leading to a reduced number of glucose-responsive *β*-like cells. Given that heterozygous inactivating mutations in *GATA6* have been linked with pancreas agenesis, these findings suggest that the severity of the phenotype could vary according to additional genetic, epigenetic, and/or environmental factors that were not accounted by the differentiation process. Interestingly, the authors also describe dosage-sensitive requirements for GATA6 and GATA4 in the formation of both definitive endoderm and pancreatic progenitor cells, confirming the complex interplays between these factors observed in genetic studies in the mouse.

In another study, Tiyaboonchai et al. used hiPSCs derived from a patient with pancreatic agenesis associated with a heterozygous *GATA6* frameshift mutation, which leads to production of a truncated protein. These researchers also used CRISPR/Cas9 genome editing to introduce this mutation on both alleles of the same hiPSC line (114). Noteworthy, hiPSC lines with homozygous mutations failed to differentiate into endoderm. Re-expression of *GATA6* or other *GATA* family members restored this defect. The use of endodermal progenitor cell lines established from the hiPSC allelic series, which expressed *GATA6* at lower levels but *GATA4* and *GATA3* at higher levels, allowed bypassing the endoderm defect and focusing on pancreatic *β* cell differentiation. The authors found that all mutant lines were able to differentiate into pancreatic *β*-like cells, but the response to glucose in these cells was functionally defective. Also, they showed that the clear decrease in pancreas specification and *β*-like cell generation was associated with limited endogenous retinoic acid signaling during *in vitro* pancreas induction using the *GATA6* mutant cell lines.

Additional information was provided by Chia et al. who combined both gene-edited and patient-derived hPSCs to study the function of GATA6 (116). These authors found that *GATA6* heterozygous hPSCs show a limited reduction in endoderm formation, while *GATA6*-null hPSCs can only form mesoderm-like cells. Thus, GATA6 seems to be upstream of the endoderm program in humans. Consistent with this hypothesis, genome-wide studies showed that GATA6 binds and cooperates with EOMES/SMAD2/3 to regulate the expression of master endoderm genes. In addition, the early deficit of GATA6^+/−^ in definitive endoderm was accompanied by a significant reduction in PDX1+ pancreatic progenitors and C-peptide+ *β*-like cells. These findings show that, in humans, the formation of definitive endoderm and acquisition of pancreatic fate are exquisitely sensitive to *GATA6* gene dosage.

Taken together, the above-mentioned reports revealed different levels of requirement of GATA6 for pancreatic differentiation between protocols, labs and cell lines (**Table 2**). In this context, a very recent report by Gadue and colleagues provides an illustrative example which might help to understand this apparent divergence. These researchers generated a hiPSC line derived from a pancreatic agenesis patient, harboring a heterozygous 4 bp duplication in exon 2 of *GATA6* leading to a premature STOP codon, a genetically matched control line, and an identically artificially-mutated ESC line. Using these cell lines the authors identified a minor allele frequency of a SNP located downstream of *GATA6* which was associated with the level of expression of this gene (117). In their *in vitro* model, the expression of the GATA6 protein remained depressed in pancreatic progenitor cells even after correction of the coding mutation. Screening the regulatory regions of the *GATA6* gene in the patient cells and an additional pancreas agenesis hiPSC line revealed the above-mentioned SNP. Noteworthy, introducing this non-coding disease modifier SNP by CRISPR/Cas9 in control hESCs confirmed that it depressed *GATA6* expression in pancreas precursors. Thus, the phenotypic diversity found in *GATA6* heterozygous patients and the outcome of *in vitro* studies could be explained in part by this genetic variant.

The findings reported by Gadue and colleagues suggest that caution has to be taken when interpreting the results of monogenic diabetes modeling using patient-derived hiPSCs. Additional genomic variants might contribute to the *in vitro* differentiation outcomes, making it difficult to compare the results obtained by different groups. Nevertheless, some of the studies mentioned above did use the same hPSC line, including the original H9 line derived by JA Thomson and colleagues (144). In such cases, it is worth to underline that each group used different protocols of differentiation. Specific additives could compensate for the decrease in *GATA6* expression. For example, retinoic acid seems to support GATA6 function in pancreatic specification. Addition/increase of this morphogen could modulate the effect of *GATA6* haploinsufficiency. Taken together, these results illustrate the challenges and, at the same time, highlight the unique interest of investigating the function of key transcription factors in pancreatic development using hPSCs.

### HNF4A

In mouse, it has been shown that full inactivation Hnf4a is embryonically lethal, while heterozygote knockout mice are normoglycemic and do not present diabetes features (145–147). In contrast, MODY1 patients carrying heterozygous mutations in *HNF4A* present diabetes due to impaired *β* cell function (148). Patient-derived hiPSCs have been recently used to address the potential mechanisms involved in this phenotype. Ræder and colleagues reported the use of hiPSCs derived from patients carrying a non-sense *HNF4A* mutation associated with MODY1 to study its effect on pancreas and *β* cell differentiation (105). Noteworthy, the mutation studied in this work (p. Ile271fs) generates a truncated HNF4A product from one of the alleles. The authors show that insulin-positive cells could be generated *in vitro* from these cells, suggesting that this human *HNF4A* mutation neither blocked the expression of the insulin gene nor the production of insulin-producing cells *in vitro*. However, they acknowledge that the insulin-producing cells derived are immature as a result of the *β* cell differentiation protocol *per se*, leaving open the possibility that HNF4A could have more subtle effects on the functionality of fully mature *β* cells.

In another study, Braverman-Gross et al. generated hiPSCs from MODY1 patients harboring a different non-sense mutation in the *HNF4A* gene and evaluated its differentiation along the pancreatic lineage (106). In this case, the mutation studied affects all *HNF4A* transcripts and impairs the protein dimerization and transactivation domains. Pancreatic progenitors differentiated from these cells exhibited an upregulation of other key pancreatic transcription factors, including *PAX6*, *NEUROD1*, and *NEUROG3*. The authors suggest that such gene expression increase could be a compensatory mechanism utilized by MODY1 cells to overcome the reduction in *HNF4A* expression. Interestingly, they also note that the differential expression of HNF4A target genes in posterior foregut progenitors derived from mutant cells is affected by the number of HNF4A DNA binding sites, its transcription start site distance, and the number of other transcription factor binding sites. Unfortunately, the authors of this work did not extend the differentiation protocol to evaluate proportion and functionality of *β*-like cells derived from these hiPSC samples.

MODY1 disease modeling was also more recently accomplished by Teo and colleagues using hiPSCs derived from patients with frameshift mutations that introduce a premature stop codon in *HNF4A*, leading to an unstable mRNA and overall lowered HNF4A levels (107). This mutation is the same one (p. Ile271fs) studied by Ræder and colleagues. Control hiPSC lines were derived from a non-diabetic patient family member. The resulting cell lines were differentiated into liver and pancreatic endocrine cells. Phenotypic analyses showed that *HNF4A* haploinsufficiency affects foregut endoderm gene expression signatures, contributing to long-term consequences on hepatic and pancreatic cell fates. While key developmental genes were perturbed by *HNF4A* haploinsufficiency at the pancreatic progenitor stage (including *HNF1B*, *PDX1*, *GATA4*, and *RFX6*), these mutant hiPSCs were still able to procure *β*-like cells expressing specific markers, including insulin and C-peptide. However, the *β*-like cells derived with the assayed *in vitro* protocol were not fully mature. More critical effects of *HNF4A* mutations taking place during the *β* cell maturation process or on already mature *β* cells could not be properly evaluated with the protocol described in this work.

Taken together, the results reported so far from hiPSC models used to study the effects of different *HNF4A* mutations suggest that the effects of such mutations might be more relevant at the functional level of the *β* cells produced. The generation of fully functional *β*-like cells from *in vitro* differentiation protocols still remains a challenge. Thus, evaluating the functionality of the *β*-like cells produced from control or patient-derived hiPSC cannot be appropriately assessed with the current differentiation protocols. On the other hand, it should be noted that while two of these studies evaluated the effects of the same mutation, the results described by Braverman-Gross et al. analyzed a different *HNF4A* mutation. These mutations lead to HNF4A loss-of-function through different mechanisms, thus potentially explaining the different outcomes obtained in each of the reports. Last, but not least, it should be kept in mind when using patient-derived hiPSCs that additional mutations in other genes or in *HNF4A* regulatory regions could also modulate the outcome of the *in vitro* differentiation experiments, as illustrated above for *GATA6*. To conclude, additional studies are necessary to address how *HNF4A* mutations cause MODY in humans, especially using the next generation of pancreatic differentiation protocols that improve the production of fully mature *β*-like cells.

### HNF1B

Teo and colleagues established a well-controlled patient-derived hiPSC pancreatic differentiation model to elucidate the molecular mechanisms underlying MODY5 pancreatic hypoplasia (113). Differentiation of MODY5-hiPSCs into pancreatic progenitors showed that the HNF1B^S148L/+^ mutation causes the up-regulation of several key endocrine pancreas-enriched transcription factors including *PDX1*. Pancreatic differentiation using these cells did not block *PDX1*, *PTF1A*, *GATA4*, and *GATA6* expression, suggesting that MODY5-mediated pancreatic hypoplasia in this case is mechanistically independent from the effect associated with these transcription factors. On the other hand, the point mutation in *HNF1B* caused an indirect reduction in the expression of the insulin gene activator *PAX6*, suggesting that loss of one copy of *HNF1B* in humans impairs *β* cell development and function. Although these findings are consistent with the potential occurrence of maturity-onset diabetes, they fail to uncover the mechanism by which *HNF1B* haploinsufficiency results in pancreatic hypoplasia.

To further address this question, we recently used an alternative hiPSC pancreatic differentiation model to elucidate the molecular mechanisms underlying HNF1B-associated diabetes (Khairi et al, manuscript submitted). To evaluate the transcriptional differences in the *HNF1B* haploinsufficient cells, we used bulk RNA-seq at several stages of the pancreatic differentiation protocol (from DE to *β*-like cells), immunofluorescence staining, and scRNA-seq at the MPC stage. Our analyses show that absence of HNF1B blocks the specification of the pancreatic fate from the foregut progenitor stage. In contrast, *HNF1B* haploinsufficiency allows differentiation of MPCs and the generation of functional *β*-like cells although at a lower frequency than the control isogenic cell line. We further report that *HNF1B* haploinsufficiency impairs cell proliferation in foregut progenitors and MPCs. Our results show that HNF1B plays a key role in the production and expansion of pancreatic progenitors and suggest that this factor could regulate the expression of several Hippo pathway components in MPCs. Thus, the level of HNF1B, combined with environmental stimuli, could define the number of pancreatic progenitor cells generated during development and therefore contribute to the susceptibility to diabetes during childhood/adulthood.

### PTF1A

It has been described that homozygous inactivating mutations in *PTF1A* cause pancreatic and cerebellar agenesis (98). In agreement, *Ptf1a* null mice present a complete absence of exocrine pancreatic tissue, but all islet endocrine cell types are present until the late stages of embryogenesis (149). Zhu et al. reported the *in vitro* modeling of the PTF1A requirement for human pancreas development. Using *PTF1A* ^−/−^ hESCs and a first generation *in vitro* pancreatic differentiation protocol, these researchers did not find defects in pancreatic endocrine differentiation (111). This finding is in agreement with previous reports showing that *Ptf1a* is not required for the specification of Ngn3+ endocrine progenitors or the differentiation of mature *β* cells in mice (150).

The study of *PTF1A* regulation provides another example of how human *in vitro* pancreatic differentiation can guide the discovery of a developmental regulatory mechanism, in this case consisting in the identification of recessive mutations in a distal non-coding region (151). Identification of genetic mutations resulting in pancreatic agenesis can be challenging as these can be located in regulatory regions far away from known regulators. Accordingly, genome sequencing of a cohort of patients presenting pancreatic agenesis revealed several mutations in a distal non-coding region located >1 Mb upstream the *PTF1A* gene. Enhancer profiling in MPCs, derived *in vitro* from hPSCs, confirmed the functional importance of this regulatory sequence in humans (151). The mutation sites coincided with a FOXA2 binding site profiled by ChIP-seq in *in vitro* MPCs. Further mechanistic experiments performed *in vitro* confirmed that the targeted region acts as an enhancer in human MPCs, and that patient mutations affect PDX1 and FOXA2 binding. These findings allowed us to propose that the mutated enhancer region is in charge of triggering the early *PTF1A* expression in the gut region where the pancreas is specified. This study illustrates how human genetic and *in vitro* differentiation of hPSCs can be combined to define mechanisms driving developmental diseases.

### INS

Balboa et al. generated a model based on hiPSCs from patients carrying *INS* mutations and engineered isogenic CRISPR-Cas9 mutation-corrected lines. These cells were differentiated to *β*-like cells (152). Using this model, the authors show that the *INS* mutations lead to accumulation of proinsulin misfolding, increased signs of ER-stress, and reduced proliferation in *INS*-mutant *β*-like cells compared with corrected controls. Following transplantation into mice, *INS*-mutant grafts presented reduced insulin secretion and further increased ER-stress, associated with decreased *PDX1* expression and *β* cell size, as well as mitochondrial alterations. The authors conclude that neonatal diabetes-associated *INS*-mutations lead to defective *β* cell mass expansion, contributing to neonatal diabetes development.

In another recent study, Egli and colleagues generated hiPSCs from fibroblasts of a patient with PNDM and undetectable insulin at birth due to a homozygous mutation in the translation start site of the insulin gene (153). Their results show that the differentiation of *INS* mutant cells resulted in hormone-negative hiPSCs, and the correction of this mutation by CRISPR-Cas9 restored insulin production and secretion to levels comparable to those of wild type endocrine cells. The authors also demonstrate that the insulin-producing cells of corrected patient hiPSCs protect mice from diabetes, providing a proof-of-principle study for the use of replacement therapy as a treatment for monogenic diabetes.

### STAT3

Saarimäki-Vire et al. used hiPSCs derived from a patient with PNDM and pancreatic hypoplasia to investigate the effects of an activating *STAT3* mutation on pancreatic development (154). Noteworthy, the mutation studied has been identified as the cause of PNDM in association with early onset autoimmunity. These authors demonstrate that the mutation in *STAT3* leads to premature endocrine differentiation through binding and direct induction of *NEUROG3* by the increased nuclear shuttling of the mutated protein. They also showed that correction of the *STAT3* mutation using CRISPR/Cas9 completely reversed the disease phenotype. These results demonstrate that, in addition to the early onset autoimmunity, the same mutation leads to a primary developmental defect in pancreatic organogenesis.

## Conclusion and Future Directions

The field of hPSCs has allowed important advances in our understanding of the molecular mechanisms underlying the different forms of monogenic diabetes. Indeed, the establishment of hPSC-based *in vitro* platforms offers a unique opportunity to study pancreas development and to investigate the pathophysiology underlying monogenic diabetes. This basic knowledge paves the way to the development of new treatments, not only for diabetes induced by genetic mutations, but also more broadly for personalized medicine therapies in the context of type I and type II diabetes. Nonetheless, several challenges require attention. Current *in vitro β*-like cell differentiation protocols have been markedly improved and may be sufficient to recapitulate several of the MODY phenotypes in the hPSC-based model. However, one of their greatest limitations remains the lack of metabolic maturation of the *β*-like cells derived. A solution to turn the differentiated cells into fully mature and functional *β* cells has been their transplantation in mouse to allow for the latest steps of cell differentiation to take place *in vivo*. Alternative methods involve culture in 3D and cell self-aggregation into islet-like clusters to produce *β*-like cells with improved functionality. The emergence of scRNA-seq is expected to lead to the identification of new markers involved in pancreatic *β* cell maturation, thus allowing improved benchmarking of the *in vitro* differentiation protocol outcomes. Also, scRNA-seq applied to human embryonic pancreatic tissue might provide additional insights into the developmental cues that differ among mice and humans. This will provide additional input to improve the *in vitro* differentiation protocols by modulating yet unknown signaling cues.

The other growing challenge is the divergence of results between different groups studying the same mutation/genes but using either different hPSC lines and/or different protocols. Indeed, genetic background and culture conditions can have a strong effect on phenotype, thus leading to different experimental outcomes. New hiPSC lines derived from monogenic diabetic patients continue to be reported. A very recent study described the generation of hiPSCs derived from MODY2 patients, but in this case its differentiation into the pancreatic lineage was not evaluated so far (108). Thus, there is a need to develop standard hiPSC lines which could be shared between laboratories. More importantly, the use of “universal” culture conditions to grow and to differentiate hPSC lines would be incredibly useful to allow the comparison of data generated and to precisely establish the importance of genetic background on the phenotype observed *in vitro*. Such standardization implies that culture conditions are fully described and shared between laboratories. The use of isogenic control hPSC lines is also essential and is helping to overcome the limitations related to the variability between lines, especially when compared with the use of family controls, which is inherent to differences in genetic background. Numerous studies have successfully used CRISPR/Cas9 tools to generate isogenic hPSC lines by introducing patient-specific mutations, editing genes in control-hPSC lines to investigate the implication of a single genetic variant on *β* cell differentiation and function. Here, we revisited the latest advances in the application of *in vitro* pancreatic cell differentiation from hPSCs to model several types of monogenic diabetes. Much work remains to be done to improve the modeling of monogenic diabetes but, as it stems from this review, *in vitro* pancreatic differentiation from hPSCs is definitely gaining momentum.

## Author Contributions

All authors reviewed the literature, wrote, and edited the paper. JIB prepared the table. LV and SAR-S conceptualized the review topic and contents, and approved the paper. All authors contributed to the article and approved the submitted version.

## Funding

This work was funded by Office of the Royal Society and the Consejo Nacional de Investigaciones Científicas y Técnicas of Argentina (IEC\R2\181023 to SAR-S and LV). Work in the Rodríguez-Seguí laboratory is supported by grants from the Agencia Nacional de Promoción Científica y Tecnológica of Argentina (ANPCyT: PICT-2015 3605, PICT-2017 2071) and the University of Buenos Aires (UBACyT 20020170200156BA). Work in the Vallier laboratory is supported by grants from European Research Council Grant New-Chol, the Cambridge Hospitals National Institute for Health Research Biomedical Research Center and core support grant from the Wellcome and Medical Research Council to the Wellcome-Medical Research Council Cambridge Stem Cell Institute.

## Conflict of Interest

The authors declare that the research was conducted in the absence of any commercial or financial relationships that could be construed as a potential conflict of interest.
